# A genomic survey of *Clostridioides difficile* isolates from hospitalized patients in Melbourne, Australia

**DOI:** 10.1128/spectrum.01352-23

**Published:** 2023-10-10

**Authors:** Sarah Larcombe, Galain C. Williams, Jacob Amy, Su Chen Lim, Thomas V. Riley, Anthony Muleta, Adele A. Barugahare, David R. Powell, Priscilla A. Johanesen, Allen C. Cheng, Anton Y. Peleg, Dena Lyras

**Affiliations:** 1 Monash Biomedicine Discovery Institute and Department of Microbiology, Monash University, Melbourne, Victoria, Australia; 2 School of Medical and Health Sciences, Edith Cowan University, Joondalup, Western Australia, Australia; 3 School of Biomedical Sciences, The University of Western Australia, Perth, Western Australia, Australia; 4 Medical, Molecular, and Forensic Sciences, Murdoch University, Perth, Western Australia, Australia; 5 Monash University, Melbourne, Victoria, Australia; 6 Department of Infectious Diseases, Alfred Hospital, Melbourne, Victoria, Australia; Griffith University--Gold Coast Campus, Gold Coast, QLD, Australia

**Keywords:** *Clostridioides difficile*, hospital-acquired infection, whole-genome sequencing, genetic epidemiology

## Abstract

There has been a decrease in healthcare-associated *Clostridioides difficile* infection (CDI) in Australia, coupled with an increase in the genetic diversity of strains isolated in these settings, and an increase in community-associated cases. To explore this changing epidemiology, we studied the genetic relatedness of *C. difficile* isolated from patients at a major hospital in Melbourne, Australia. Whole-genome sequencing of *C. difficile* isolates from symptomatic (*n* = 61) and asymptomatic (*n* = 10) hospital patients was performed. Genomic comparisons were made using single-nucleotide polymorphism (SNP) analysis, ribotyping, and toxin, resistome, and mobilome profiling. *C. difficle* clade 1 strains were found to be predominant (64/71), with most strains (63/71) encoding both toxins A and B (A+B+). Despite these similarities, only two isolates were genetically related (≤2 SNPs) and a diverse range of ribotypes was detected, with those predominating including ribotypes commonly found in community-associated cases. Five non-toxigenic (A−B−CDT−) clade 1 strains were identified, all in asymptomatic patients. Three clade 4 (A−B+CDT−) and four clade 5 (A+B+CDT+) strains were detected also, with these strains more likely to carry antimicrobial resistance determinants, many of which were associated with mobile genetic elements. Overall, within a single hospital, *C. difficile*-associated disease was caused by a diverse range of strains, including many strain types associated with community and environmental sources. While strains carried asymptomatically were more likely to be non-toxigenic, toxigenic strains were isolated also from asymptomatic patients, which together suggest the presence of diverse sources of transmission, potentially including asymptomatic patients.

**IMPORTANCE:**

There has been a decrease in healthcare-associated *Clostridioides difficile* infection in Australia, but an increase in the genetic diversity of infecting strains, and an increase in community-associated cases. Here, we studied the genetic relatedness of *C. difficile* isolated from patients at a major hospital in Melbourne, Australia. Diverse ribotypes were detected, including those associated with community and environmental sources. Some types of isolates were more likely to carry antimicrobial resistance determinants, and many of these were associated with mobile genetic elements. These results correlate with those of other recent investigations, supporting the observed increase in genetic diversity and prevalence of community-associated *C. difficile*, and consequently the importance of sources of transmission other than symptomatic patients. Thus, they reinforce the importance of surveillance for in both hospital and community settings, including asymptomatic carriage, food, animals, and other environmental sources to identify and circumvent important sources of *C. difficile* transmission.

## INTRODUCTION

*Clostridioides difficile* is the primary aetiological agent of infectious diarrhea in hospitalized patients (1), with infection remaining a significant global problem in healthcare facilities worldwide (2, 3). However, both the clinical and molecular epidemiology of *C. difficile* infection (CDI) have changed over time and, while community-associated cases are increasing, there is now a decrease in healthcare-associated cases. These cases are associated with increasingly genetically diverse strains (4).

*C. difficile* can transiently (or longer in hospital patients) colonize the gut asymptomatically or cause a spectrum of diarrheal diseases collectively known as CDI, mediated by two major toxins TcdA (toxin A) and TcdB (toxin B). Some strains also encode an additional binary toxin known as (*C. difficile* transferase (CDT) (5). Variant strains carrying different combinations of these three toxins or containing variations within the toxin genes are seen also (6, 7). The spread of *C. difficile* is facilitated by the production of highly resistant spores, which are shed in the feces of infected individuals and are thought to be the major source of transmission (8). However, infection prevention and control measures, and antimicrobial stewardship in healthcare facilities have been successful in preventing transmission of *C. difficile* between symptomatic patients, and the genetic diversity of strains isolated in these settings has increased (4, 9 – 13). This genetic diversity mirrors that seen in community-associated CDI, supporting the importance of infectious sources other than symptomatic patients. Asymptomatic carriers of toxigenic *C. difficile* strains have been suggested as an alternative source of infection, and transmission between community and healthcare settings has been proposed also, with a recent study showing that 79% of *C. difficile* ribotypes detected in Australian hospitals were detected also in the community (14). Therefore, the identification and molecular typing of *C. difficile* strains carried by both asymptomatic carriers and symptomatic CDI patients within healthcare facilities may provide important information about the epidemiology of CDI and inform infection prevention and control practices.

During a recent study at a Melbourne hospital, 71 *C*. *difficile* isolates were recovered from patients who presented with CDI symptoms (*n* = 61), or those who did not (*n* = 10). Subsequently, we investigated the genetic relatedness of these strains with PCR ribotyping and analysis of whole-genome sequence data using *in silico* multi-locus sequence typing (MLST), and further characterisation based on antimicrobial resistance (AMR) determinants and mobile genetic elements (MGEs). This investigation provides important information on circulating strains in a large Australian hospital. The findings support previous work suggesting that strains causing healthcare-associated CDI within a single facility are genetically distinct outside of an outbreak setting, and propose the presence of important sources of infection other than symptomatic patients.

## MATERIALS AND METHODS

### Study design and participants

From 2014 to 2015, a *C. difficile* surveillance study was conducted at a >500-bed public hospital in Melbourne, Australia. Patients were included if they had *C. difficile* isolated from stool; current clinical guidelines recommend testing all patients with diarrhea who are inpatients. Standard laboratory criteria rejected non-liquid stool, except where specifically requested by the clinician due to a high clinical suspicion of CDI. For this study, faecal samples were collected in sterile containers and tested for *C. difficile* toxin using *C. difficile* Tox A/B II (TechLab). Isolates described as asymptomatic were recovered from formed stools from patients without CDI across multiple wards and floors from the same public hospital. *C. difficile* was isolated from enzyme immunoassay positive samples using ChromID *C. difficile* Agar (bioMérieux) and cultured for storage in cooked meat medium.

### Bacterial culture

For the extraction of genomic DNA, *C. difficile* from cooked meat medium stocks was cultured anaerobically at 37°C in supplemented Heart Infusion (HIS) broth or on agar plates (Oxoid), supplemented with 0.375% glucose, 0.1% cysteine, and 0.1% sodium taurocholate.

### Isolation of genomic DNA and sequencing

Genomic DNA was isolated from *C. difficile* as described previously (15). Whole-genome sequencing was performed on the Illumina MiSeq Platform, as described previously (16).

### Bioinformatic analysis

QC, assembly, annotation, and analysis of each genome were performed using the Nullarbor v2.0 pipeline (https://github.com/tseemann/nullarbor). *C. difficile* strain 630 (RT012, ST54) was used as the reference genome for all analyses performed with Nullabor (NCBI GenBank Accession no. NC_009089). Details on the assembled draft genomes are detailed in Table S1. Phylogenetic analysis was performed through the Nullarbor pipeline with the default settings using IQ-Tree V1.6,9 (17) and FastTree V2.1.10 (18) for the core and pan-genome phylogeny, respectively. Detection of virulence factors and MGEs was performed using Nullabor with ABRicate (https://github.com/tseemann/abricate) on default settings against the following databases: NCBI AMRFinderPlus (19), CARD (20), ResFinder (21), ARG-ANNOT (22), VFDB (23), PlasmidFinder (24), and MEGARes 2.0 (25). For further detection of *C. difficile* toxins, and other MGEs, NCBI BLASTn was used against reference gene sequences (Table S2) with a threshold of e^−10^ for positive hits, and multiple sequence alignments were performed using EBI-EMBL Clustal Omega (https://www.ebi.ac.uk/Tools/msa/clustalo/).

### PCR ribotyping

Purified genomic DNA, as used for sequencing, was used as the PCR template. PCR ribotyping was performed as previously described (26, 27). PCR ribotyping reaction products were concentrated using the Qiagen MinElute PCR purification kit (Qiagen Sciences, Germantown, MD, USA) and run on the QIAxcel capillary electrophoresis platform (Qiagen Sciences). The analysis of PCR ribotyping products was performed using the BioNumerics software package, v.7.6.3 (Applied Maths, Sint-Martens-Latem, Belgium). PCR RTs were identified by comparison with banding patterns in a reference library, consisting of a collection of 80 reference strains from the European Centre for Disease Prevention and Control and the most prevalent RTs currently circulating in Australia and Asia (T. V. Riley, unpublished data). Isolates that could not be identified with the reference library were designated with internal nomenclature, prefixed with QX, if there were more than two isolates with the same banding pattern. A single isolate with a new banding pattern was designated “novel.”

## RESULTS

### Isolation and whole-genome sequencing of *C. difficile* from hospitalized patients

During the sample period, 71 *C*. *difficile* isolates were recovered using faecal swabs from patients with (*n* = 61) or without (*n* = 10) symptomatic CDI. Following the recovery of *C. difficile* from patient samples, genomic DNA was extracted for PCR ribotyping and whole-genome sequencing. *In silico* analyses of draft genome sequences included single-nucleotide polymorphism (SNP) analysis and core genome phylogeny, determining multi-locus sequence type (MLST), toxin gene profiling, pan-genome phylogeny, and resistome and mobilome analysis (Table 1).

**TABLE 1:** The mobilome and resistome of hospital-associated *C. difficile* isolates^*a*^

Isolate type/strain no.	Mobile genetic elements			Antibiotic resistance determinants			Classification	
	Plasmids	Bacteriophage	Transposons	Resistance genes	GyrA mutations	GyrB mutations	RT	ST
Symptomatic								
MCD1						I139R	014/020	2
MCD2						I139R	012/020	55
MCD4			Tn*6086*	*tet*(M)			012	54
			Tn*6218*	*aacA*/*aphD, erm*(B)				
MCD5						I139R	012/020	2
MCD6	pCD6						002	8
MCD7						I139R	012/020	2
MCD8	pCD6						002	8
MCD10	pCDBI1						novel	103
MCD11						I139R	012/020	2
MCD12						I139R	012/020	2
MCD14						I139R	012/020	2
MCD15						I139R	012/020	2
MCD16			Tn*5397*	*tet*(M)		**S366A**	221	38
MCD17						I139R	012/020	13
MCD18	pCDBI1		Undefined MGE^*b*^	*tet*(O)		**S366A**	054	43
MCD19						I139R	012/020	13
MCD21						I139R	012/020	2
MCD22	pCDBI1		Undefined MGE^*b*^	*tet*(O)		**S366A**	054	43
MCD23			Tn*916*	*tet*(M)	K413N	Q160H, **S366V**, **S416A**	Novel	11
			Tn*6218*	*cfrB*				
			Tn*6194*	*erm*(B)				
			Tn*6189*-like	*aadE-sat4-aphA-3*				
			Undefined MGE^*b*^	*tet*(40), *aadE*-like				
				*aad9^c^*				
MCD24						I139R	012/020	2
MCD28			Tn*916*	*tet*(M)			Novel	129
			Tn*6218*	*aacA*/*aphD, erm*(B)				
MCD30						**S366A**	018	17
MCD33			Tn*6194*	*erm*(B)		**S366A**	018	17
MCD36						**S366A**	054	43
MCD38	pCDBI1						157	234
MCD39	pCD6		Tn*916*	*tet*(M)		I139R	012/020	49
			Tn*6194*	*erm*(B)				
MCD40			Tn*5801*-like	*tet*(M)			012	54
			Tn*6218*	*aacA*/*aphD, erm*(B)				
MCD41		φCD38-2			G490C	**S366A**	018	17
MCD42	pCD6					I139R	014/020	14
MCD43	pCD6		Undefined MGE^*b*^	*tetA*(*P*), *tetB*(*P*)		I139R	012/020	2
MCD44	pCD6, pDLL3026						002	8
MCD46	pCD6		Undefined MGE^*b*^	*tetA*(*P*), *tetB*(*P*)		I139R	012/020	2
MCD49	pDLL3026						002	8
MCD52						**S366A**	054	43
MCD53						V130I	015	44
MCD55		φCD38-2			G490C	**S366A**		17
MCD57						I139R	012/020	14
MCD58	pCD6						103	53
MCD60						I139R	012/020	13
MCD61	pCD6					I139R	012/020	2
MCD66	pCD6						002	8
MCD67	pCD630		Tn*916*	*tet*(M)	**T82I**, K413N	Q160H, **S366V**, **S416A**	Novel	11
			Tn*6194*	*erm*(B)				
			Tn*6189*-like	*aadE-sat4-aphA-3*				
			Tn*4453a*/*b*-like	*aph2-aadA-aac-aacA*/*aphD*				
			Undefined MGE^*b*^	*tet*(40), *aadE*-like				
				*aad9* **^*c*^**				
MCD68			Tn*916*	*tet*(M)	**T82I**	**S366A**	017	37
			Tn*6218*	*cfr*(B)				
			Undefined MGE^*b*^	*aacA*/*aphD*				
				*aadE*-like^*c*^				
MCD70			Tn*916*	*tet*(M)	**T82I**, K413N	Q160H, **S366V**, **S416A**	Novel	11
			Tn*6194*	*erm*(B)				
			Tn*4453a*/*b*-like	*aph2-aadA-aac-aacA*/*aphD*				
			Tn*6189*-like	*aadE-sat4-aphA-3*				
			Undefined MGE^*b*^	*tet*(40), *aadE*-like				
				*aad9^c^*				
MCD71	pDLL3026						002	8
MCD72						I139R	012/020	14
MCD73						**S366A**	284	80
MCD74						**S366A**	284	80
MCD77	pCD6						002	8
MCD79			Tn*916*	*tet*(M)			Novel	129
			Tn*6218*	*aacA*/*aphD, erm*(B)				
MCD83			Tn*916*	*tet*(M)	**T82I**	**S366A**	017	37
			Tn*6218*	*cfrB*				
			Undefined MGE^*b*^	*aacA*/*aphD*				
				*aadE*-like^*c*^				
Total symptomatic isolates = 61								
Asymptomatic isolates								
MCD26							Novel	101
MCD27							010	15
MCD29			Undefined MGE^*b*^	*tet*(M), *cfrC*			081	9
			Tn*6189*	*erm*(B)				
MCD31			Tn*6189*	*erm*(B)			Novel	107
MCD45	pCD6, pCDBI1							8
MCD47			Tn*916*	*tet*(M)			Novel	129
			Tn*6218*	*aacA*/*aphD, erm*(B)				
MCD48			Tn*916*	*tet*(M)	K413N	Q160H, **S366V**, **S416A**, **E466V**	Novel	11
			Undefined MGE^*b*^	*tet*(O)				
			Undefined MGE^*b*^	tet(40), *aadE*-like				
			Tn*6189*-like	*aadE-sat4-aphA-3*				
MCD51							010	15
MCD64			Tn*5397*	*tet*(M)	M324I	V130I	046	35
			Tn*6218*	*aacA*/*aphD, erm*(B)				
			Tn*4453*a	*catD*				
				*aadE*-like**^*c*^**				
MCD80			Tn*5397*	*tet*(M)			039	26
			Undefined MGE^*b*^	*erm*(B)				
			Undefined MGE^*b*^	*aph*(2")-If				
				*aadE*-like^*c*^				
Total asymptomatic strains = 10								

^*a*^ The detection of mobile genetic elements including transposons, plasmids, and bacteriophage was performed using NCBI BLASTn against reference gene sequences, and the presence of antibiotic resistance genes was predicted using the Nullarbor v2.0 pipeline (https://github.com/tseemann/nullarbor). MLST STs were determined using the Nullarbor pipeline according to the pubMLST database (https://pubmlst.org/organisms/clostridioides-difficile). Antibiotic resistance genes associated with transposons are listed in the same row. Amino acid substitutions in GyrAB proteins were detected by multiple sequence alignments were performed using EBI-EMBL Clustal Omega (https://www.ebi.ac.uk/Tools/msa/clustalo/), in comparison to the fluoroquinolone-susceptible reference strain *C. difficile* 630. GyrAB mutations previously reported to confer fluoroquinolone resistance are in bold.

^*b*^ Undefined element containing transposon-like genes.

^*c*^ Genomic location undetermined.

### Molecular epidemiology and relatedness of hospital-associated *C. difficile* isolates

**Fig 1:**
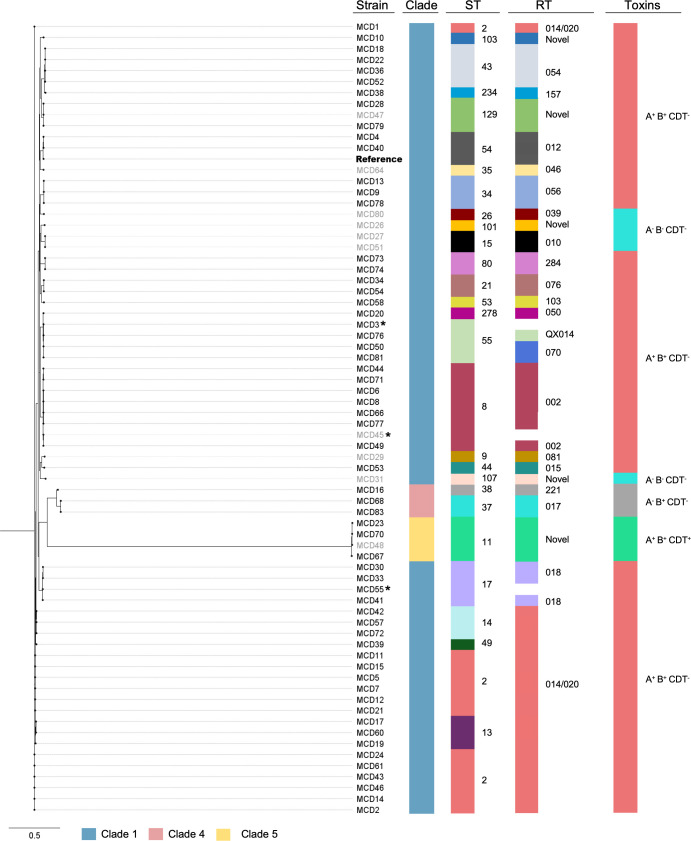
Core phylogenetic analysis, MLST, ribotyping, and toxin profiling of hospital-associated *C. difficile* isolates. Using the Nullabor v2.0 pipeline (https://github.com/tseemann/nullarbor), a core genome phylogeny of 99,370 SNPs was inferred with a maximum-likelihood tree using a GTR + G4 model, and multi-locus sequence types and clades were determined according to the *C. difficile* pubMLST database (https://pubmlst.org/organisms/clostridioides-difficile). Ribotypes were determined via PCR ribotying, and toxins were detected *in silico* using NCBI BLASTn against reference gene sequences. The phylogenetic analysis was performed in comparison to the reference strain, *C. difficile* 630, denoted here in bold. The isolates detected in symptomatic CDI patients are denoted in black, and those detected in asymptomatic patients are denoted in gray. Isolates marked with an asterisk (*) could not be ribotyped. Each isolate is represented on the phylogenetic tree in relation to their clade, multi-locus sequence type (ST), ribotype (RT), and encoded toxins (toxins A, B, and CDT) with the + and – symbols denoting the presence or absence, respectively, of these toxin genes *in silico*. Each color represents the different groups detected in each typing scheme as a visual representation of diversity. Scale bar = substitutions per site.

**Fig 2:**
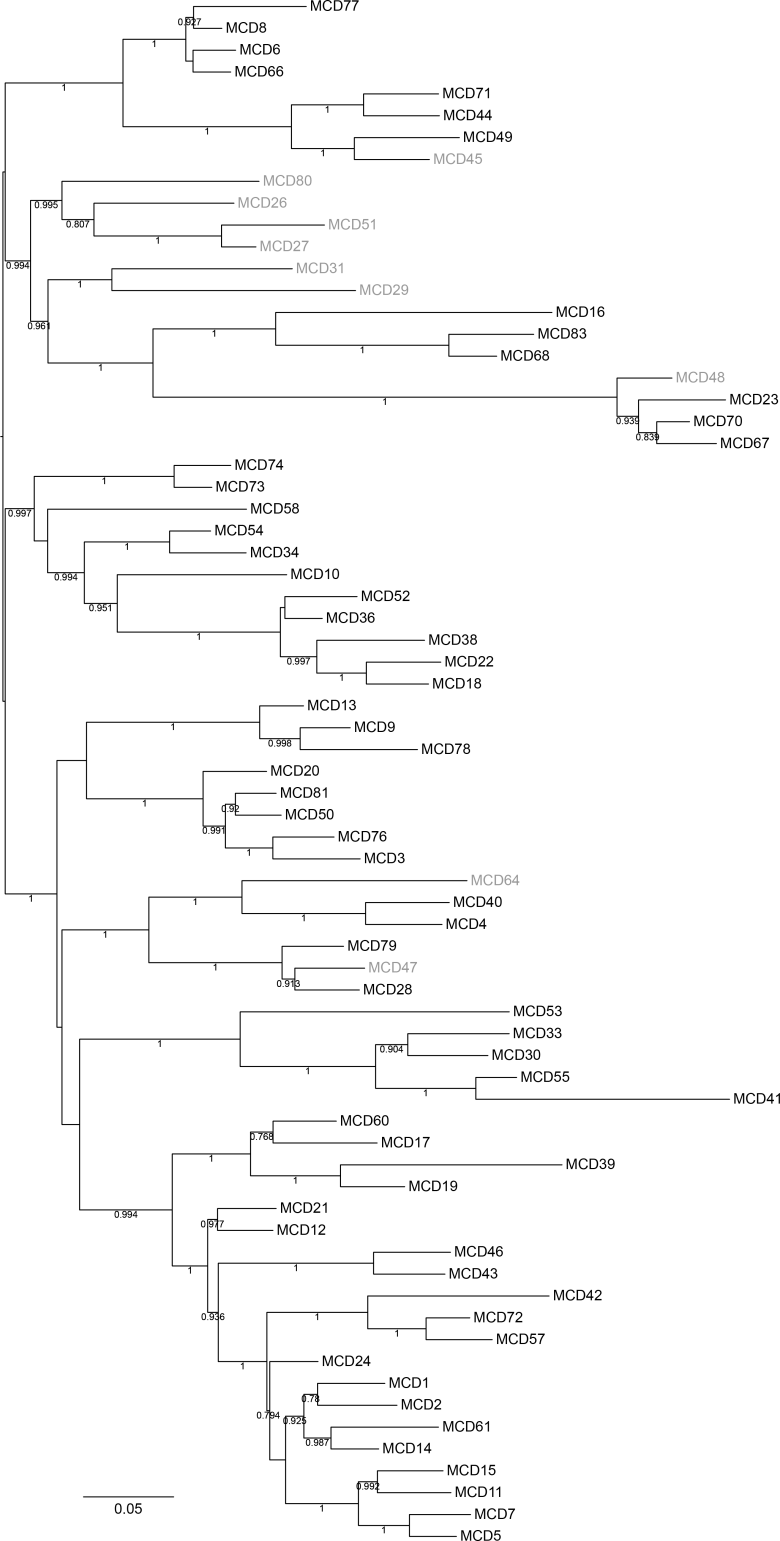
Pan-genome phylogeny of hospital-associated *C. difficile* isolates. A maximum-likelihood tree was inferred using a GTR model for the accessory genome (9,369 genes total) which was determined by the presence or absence of any non-singleton genes using the Nullabor v2.0 pipeline (https://github.com/tseemann/nullarbor). Local support values depicted against each branch were calculated with 1,000 resamples and the Shimodaira-Hasegawa test which was performed with FastTree (18). The midpoint root for the tree was estimated using iTol and tree annotation was also performed in iTol (28). The isolates detected in symptomatic CDI patients are denoted in black, and those detected in asymptomatic patients are denoted in gray. Scale bar = substitutions per site.

Phylogenomic analyses were performed on draft genome sequences to compare the evolution and genetic relatedness of individual isolates. Of the 71 isolates, 64 (90%) fell within the clade 1 phylogenetic lineage, 4 (6%) within clade 5 (all ST11), and 3 (4%) within clade 4 (ST37 and ST38) (Fig. 1). Among the clade 1 isolates, there were 24 unique sequence types, with ST2 (14/64) and ST8 (8/64) being the most predominant (Fig. 1). The predominance of clade 1 did not differ between isolates from symptomatic (55/61) or asymptomatic patients (9/10), though ST2 was not present amongst isolates from asymptomatic patients, and instead sequence types ST129 (1/10), ST35 (1/10), ST26 (1/10), ST101 (1/10), ST15 (2/10) ST8 (1/10), ST9 (1/10), ST107 (1/10), and ST11 (1/10) were observed (Fig. 1). SNP analysis of the core genome (1,734 genes and 99,370 SNPs in total) revealed that out of the 71 isolates analyzed most isolates differed by greater than >10 SNPs, with the greatest difference to the reference strain *C. difficile* 630 belonging to MCD70 (68,607 SNPs). A small number of isolates showed a high degree of genetic similarity, two (MCD4 and MCD40) had ≤2 SNPs difference, both belonging to clade 1, ST54. Four isolate pairs had between 3 and 10 SNPs difference, comprising a total of five individual isolates, with three of these isolates (MCD28, MCD47, and MCD79) belonging to clade 1 (ST129) and the remaining two (MCD57 and MCD72) belonging to clade 1 (ST14). Four isolates belonging to clade 5 (MCD70, MCD67, MCD48, and MCD23) had >60,000 SNPs in comparison to the reference strain *C. difficile* 630, though between themselves had <400 SNPs. This is reflected in a phylogenetic tree generated from core genome SNP data (Fig. 1) which showed a high degree of conservation between the reference strain, *C. difficile* 630, and most of the clinical isolates, except for a subtree containing the seven isolates belonging to clades 4 and 5 (MCD16, MCD23, MCD48, MCD67, MCD68, MCD70, and MCD83). As expected, the pan-genome analysis (9369 genes) revealed a more distant relationship than observed in the core genome (Fig. 2).

PCR ribotyping has been the reference typing method for *C. difficile* for many years, and therefore was performed in addition to *in silico* typing methods. The 71 isolates collected in this study belonged to 19 unique ribotypes (Fig. 1). The most well-represented ribotype was 014/020 (clade 1, STs 2, 13, 14, and 49), with 19/71 (27%) isolates falling within this group. Other common ribotypes included 002 (7/71; clade 1, ST8), and 054 (4/71; clade 1, ST 43). There were also 12 isolates identified that belonged to seven novel ribotypes, including the four clade 5 (ST11) isolates (Fig. 1).

### Toxin gene profiles of hospital-associated *C. difficile* isolates

Genetic markers of virulence may be useful predictors of disease severity, thus all genome sequences were analyzed for the presence of genes encoding toxins A and B, and CDT. This analysis revealed that 63 of the 71 isolates (89%) encoded both major toxins (A+B+), with 3 (4%) encoding toxin B only (A−B+), and the remaining 5 (7%) being non-toxigenic (A−B−) (Fig. 1). All three A−B+ isolates belonged to clade 4 (STs 37 and 38) and were recovered from the 61 patients with symptomatic CDI (5%). There were also four A+B+ isolates that were CDT+, with all four belonging to clade 5 (ST11). Three of the A+B+CDT+ isolates were isolated from patients with symptomatic CDI (5%) (Fig. 1). The remaining A+B+CDT+ isolate was isolated from 1 of the 10 asymptomatic patients as were the five non-toxigenic isolates all belonging to clade 1. The remaining four isolates in this patient group were A+B+CDT− and belonged to clade 1 (Fig. 1).

### The resistome of hospital-associated *C. difficile* isolates

The presence of AMR determinants was assessed *in silico*, with approximately 31% of isolates (22/71) predicted to encode AMR genes (Table 1), including approximately 23.5% of clade 1 isolates (15/64), and all clade 4 (3/3) and clade 5 (4/4) isolates. About 70% of isolates from asymptomatic patients encoded a predicted AMR gene (7/10), and 24.5% from symptomatic patients did (15/61). The predicted genes identified included the aminoglycoside resistance loci *aadE-sat4-aphA-3* and *aph2-aadA-aac-aacA*/*aphD*, the chloramphenicol resistance gene *catD*, the erythromycin resistance gene *erm*(B) and the tetracycline resistance genes *tet*(40), *tetA*(*P*), *tetB*(*P*), and *tet*(M) (Table 1).

Amino acid substitutions in the DNA gyrase proteins GyrA and GyrB that may confer resistance to fluoroquinolone antimicrobials were also analyzed. Approximately 12.5% (9/71) of isolates carried amino acid substitutions in GyrA, including all clade 5 isolates (4/4), two clade 4 isolates (2/3), and three clade 1 isolates (3/64) (Table 1). Four unique substitutions in GyrA were detected, including one that has been reported to confer resistance to fluoroquinolones in *C. difficile* (T82I) (29), which was found here in clades 4 and 5 isolates only (Table 1). All isolates that carried a mutation in GyrA also carried one in GyrB (9/71), with 55% (39/71) of isolates carrying amino acid substitutions in GyrB, including 50% of clade 1 isolates (32/64), and all clade 4 (3/3) and clade 5 (4/4) isolates (Table 1). Of note, the GyrB sequence for clade 4 isolate MCD16 was incomplete; therefore, the presence of some GyrB mutations in this isolate was not able to be determined.

### The mobilome of hospital-associated *C. difficile* isolates

MGEs are an important component of the *C. difficile* genome, with up to 11% of the genome originating from exogenous elements, and the associated insertions and recombination events driving evolution of the *C. difficile* genome (30, 31). Therefore, the genome sequence of each isolate was probed for MGEs previously identified in *C. difficile*, finding that MGEs were carried by approximately 52% (37/71) of isolates in this study, including approximately 47% (30/64) of clade 1 isolates, and all clade 4 (3/3) and clade 5 (4/4) isolates (Table 1). The detection of transposons primarily focussed on those known to encode AMR determinants; however, all identified resistance genes were also analyzed for transposon-related genes up- and downstream. Known transposons and novel transposon-like elements were detected in approximately 31% (22/71) of isolates in this study, with the presence of each being successfully correlated with resistance genes predicted *in silico* (Table 1). Known and predicted transposons were detected in 26% (16/61) of isolates from symptomatic patients and 60% (6/10) of isolates from asymptomatic patients (Table 1). Ten isolates carrying Tn*916*-like elements, and three isolates carrying Tn*5397* were predicted to encode *tet*(M), one isolate carrying Tn*4453* was predicted to encode *catD*, and five isolates carrying Tn*6194* were predicted to encode *erm(B)* (Table 1). Four isolates were predicted to encode the *aadE-sat4-aphA-3* aminoglycoside resistance locus on a novel Tn*6189*-like element, whereas it appears that the region usually encoding *ermB* had been replaced with a region encoding *aadE-sat4-aphA-*3 (Table 1), with conserved flanking regions showing up to 96% nucleotide identity (data not shown). Similarly, two clade 5 isolates carrying a Tn*4453a/b*-like transposon were predicted to encode the aminoglycoside resistance locus, *aph2-aadA-aac-aacA*/*aphD*, which also appears to have replaced a region usually encoding *catD* (32).

All genomic sequences were also queried for the presence of known *C. difficile* plasmids, including pCD630 and pCD6 (30, 33), pDLL3026 (16, 30, 31, 33), and pCDBI1 (31). These plasmids were detected in approximately 27% (19/71) of all isolates in this study, with 12 isolates carrying pCD6, 1 carrying pCD630, and 8 isolates carrying plasmids with identity to pDLL3026 and pCDBI1 (Table 1).

This analysis also included examination for *C. difficile* bacteriophages with a predicted role in virulence, including ϕCD38-2, ϕCD27, and ϕCD119, which have been implicated in the regulation of TcdA and TcdB (34). Only two isolates (2/71) were predicted to carry a bacteriophage, with both encoding the ϕCD38-2 prophage (Table 1).

## DISCUSSION

An epidemiological survey for the presence of *C. difficile* in patients admitted to a Melbourne hospital was conducted, in which 71 isolates of *C. difficile* were recovered from symptomatic (*n* = 61) and asymptomatic (*n* = 10) patients. All isolates were whole-genome sequenced, with phylogenetic analysis showing that the circulating strains in this single facility were genetically diverse, and thus unlikely to have been transmitted between symptomatic inpatients. Most of the isolates (64/71) fell within the clade 1 phylogenetic lineage, a highly heterogeneous group, with the remaining isolates belonging to clade 4 (3/71) and clade 5 (4/71) (Fig. 1). This result is in line with recent studies showing an epidemiological shift in healthcare-associated CDI, with high levels of genetic diversity detected within hospitals (4, 13, 14), rather than single lineages dominating the hospital environment as seen in outbreaks.

The genetic diversity of isolates detected in this study also supports the likelihood of a multitude of infection sources other than symptomatic patients, including asymptomatic carriers, food, and the environment (4, 9 – 11, 13, 14, 35 – 40). Similarly, high levels of genetic diversity between *C. difficile* strains isolated from symptomatic CDI patients have previously been reported (41), though the findings of this study indicate that this diversity is recapitulated even in patients not suffering from symptomatic CDI. Further supporting the divergence in infection sources are the increasingly blurred lines between strain types that cause healthcare-acquired versus community-associated CDI. Of note, the most prevalent ribotypes detected in hospitalized patients in this study were commonly associated with community-associated infection and environmental contamination, including the clade 1 (A+B+CDT−) ribotypes 014/020 (19/71) and 002-like (7/71), two of the most frequently isolated ribotypes in Australia (14, 35) (Fig. 1). *C. difficile* ribotype 014/020 strains are commonly isolated from both hospital-acquired and community-associated CDI cases in Australia, as well as being detected in asymptomatic patients (14, 36), and environmental sources such as public lawns (39), soil and mulch from hospital grounds (40), and retail root vegetables (38). *C. difficile* 014/020 strains are also commonly isolated from pigs in Australia (37, 42), with high levels of genetic relatedness between pig and human isolates, suggesting that transmission occurs between species (37). *C. difficile* ribotype 002 strains are often associated with community-associated CDI in Australia (14, 36), and can also be detected in environmental sources such as public lawns (39) and root vegetables (38). Similar trends have been seen in the UK, where one study showed that *C. difficile* isolated from patients with CDI within a single hospital tends to be genetically diverse, however highly related strain types can be isolated from environmental sources in the same geographic region (13). Therefore, it is possible that sources such as animals, food, and the environment have contributed to the spread of these lineages throughout Australia, leading to their inevitable prevalence in hospitalized patients.

Epidemic ribotypes that are commonly isolated in the Northern Hemisphere such as the clade 2 ribotype 027 and the clade 5 ribotype 078 are not endemic in Australia and were not detected in this study (Fig. 1). However, similar clade 5 ribotypes such as ribotype 126—which is closely related to ribotype 078—and ribotypes 127 and 033 are found in Australian livestock (27). This was reflected in this study, although a novel ST11 strain, ES1368 (A+ B+CDT+) made up 5.6% (4/71) of the strains detected (Fig. 1), suggesting further genetic diversity within this clinically important clade. These clade 5 strains were also the only strains found in this study to encode the *C. difficile* binary toxin (CDT) (Fig. 1) which is associated with “hypervirulent” lineages. Unsurprisingly, all isolates recovered from symptomatic CDI patients in this study encoded toxin B (Fig. 1), consistent with previous findings that toxin B is essential for virulence in *C. difficile* (43). Further supporting the role of toxin B in CDI pathogenesis is the increasing prevalence of strains encoding toxin B but not toxin A (7, 44, 45), three of which were identified in this study in symptomatic CDI patients (Fig. 1). These toxin-variant clade 4 (A−B+CDT−) strains made up 4.2% (3/71) of the total isolates detected in this study, with two of these clade 4 isolates belonging to ribotype 017 (Fig. 1). Clade 4 ribotype 017 strains are predominant in Asia (46), and their low prevalence in Australia, as seen in this and previous studies, suggests that these cases may be caused by imported, rather than circulating strains (46). While asymptomatic patients had a higher rate of non-toxigenic *C. difficile* carriage in this study (Fig. 1), the presence of toxigenic *C. difficile* in this patient group is consistent with a recent meta-analysis showing toxigenic strains present upon admission in asymptomatic patients ranging in prevalence from 4.1% to 15% (12), suggesting that asymptomatic carriage of toxigenic *C. difficile* may represent an important source of transmission within hospitals and the community. However, the relatively few *C. difficile* detected in asymptomatic patients (*n* = 10) compared to symptomatic patients (*n* = 61) in this study limits our ability to make comparisons between these patient groups. This limitation was also evident in the analysis of MGE carriage in this study, which found a higher prevalence in the smaller asymptomatic patient group (60% vs 26% in symptomatic patients).

*C. difficile* is known as one of the most urgent threats to public health due to its antimicrobial resistance (47), and the carriage of MGEs encoding AMR genes in this study sees *C. difficile* acting as a potential reservoir of these resistance genes in the gut (Table 1). While there is an established link between AMR and CDI outbreaks (1), the isolates recovered in this study were determined to be genetically distinct, with no indication of a *C. difficile* outbreak being responsible for infections. However, the abundance of AMR determinants carried by the isolates in this study, particularly in clades 4 and 5 (Table 1), suggests that antimicrobial use contributes to the persistence of these lineages. Determination of antibiotic resistance susceptibility in these clades 4 and 5 isolates may provide further insight into the functional role of the detected genes in the persistence of these isolates. Of note, there were seven unique amino acid substitutions detected in GyrB in this study, four of which have been previously associated with fluoroquinolone resistance in *C. difficile* (S366A, S366V, S416A, and E466V) (48 – 50); however, Australia has largely avoided the clonal spread of *C. difficile* associated with fluoroquinolone use in the Northern Hemisphere, likely because of the conservative use of these antimicrobials in Australia (51).

Overall, the results of this study correlate with those of other recent investigations (9 – 14), supporting the observed increase in genetic diversity and prevalence of community-associated strain types amongst *C. difficile* within a single hospital setting in Australia, and consequently, the apparent importance of sources of transmission other than symptomatic patients. Further surveillance is required for *C. difficile* in both hospital and community settings, including asymptomatic carriage, food, animals, and other environmental sources, to identify and circumvent important sources of *C. difficile* transmission.

## SUPPLEMENTAL MATERIAL

Supplemental tables spectrum.01352-23-s0001.docx Tables S1 and S2.

ASM does not own the copyrights to Supplemental Material that may be linked to, or accessed through, an article. The authors have granted ASM a non-exclusive, world-wide license to publish the Supplemental Material files. Please contact the corresponding author directly for reuse.
