# Supplementary material for: A genomic survey of Clostridioides difficile isolates from hospitalized patients in Melbourne, Australia
Source: Microbiol Spectr. 2023 Oct 10;11(6):e01352-23. doi: 10.1128/spectrum.01352-23 (PMC10715045; doi:10.1128/spectrum.01352-23)
Supplement: Supplemental tables — Tables S1 and S2. [file spectrum.01352-23-s0001.docx]

Table 1: Assembly data for draft genomes

| **Isolate** | **Contigs** | **bp** | **N50** | **Accession** |
| --- | --- | --- | --- | --- |
| MCD1 | 462 | 4045368 | 15097 | JASSYW000000000 |
| MCD10 | 349 | 4034048 | 20974 | JASSYV000000000 |
| MCD11 | 464 | 4071380 | 15717 | JASSYU000000000 |
| MCD12 | 301 | 3967621 | 25418 | JASSYT000000000 |
| MCD13 | 414 | 3986821 | 17616 | JASSYS000000000 |
| MCD14 | 357 | 4049709 | 21790 | JASSYR000000000 |
| MCD15 | 438 | 4076116 | 17816 | JASSYQ000000000 |
| MCD16 | 1048 | 4297263 | 7160 | JASSYP000000000 |
| MCD17 | 345 | 4184660 | 20718 | JASSYO000000000 |
| MCD18 | 485 | 4210808 | 16000 | JASSYN000000000 |
| MCD19 | 286 | 4149353 | 24817 | JASSYM000000000 |
| MCD2 | 541 | 4034724 | 11849 | JASSYL000000000 |
| MCD20 | 224 | 4019496 | 38599 | JASSYK000000000 |
| MCD21 | 332 | 3965117 | 22029 | JASSYJ000000000 |
| MCD22 | 512 | 4199597 | 16014 | JASSYI000000000 |
| MCD23 | 398 | 4063084 | 18409 | JASSYH000000000 |
| MCD24 | 273 | 4030518 | 26140 | JASSYG000000000 |
| MCD26 | 355 | 4021198 | 21942 | JASSYF000000000 |
| MCD27 | 304 | 3948485 | 28430 | JASSYE000000000 |
| MCD28 | 381 | 4054465 | 18853 | JASSYD000000000 |
| MCD29 | 535 | 4228169 | 14704 | JASSYC000000000 |
| MCD3 | 517 | 3985638 | 13022 | JASSYB000000000 |
| MCD30 | 333 | 4239449 | 23494 | JASSYA000000000 |
| MCD31 | 280 | 4195896 | 28207 | JASSXZ000000000 |
| MCD33 | 301 | 4341175 | 25158 | JASSXY000000000 |
| MCD34 | 367 | 4014793 | 19898 | JASSXX000000000 |
| MCD36 | 321 | 4079421 | 21791 | JASSXW000000000 |
| MCD38 | 445 | 4096963 | 18000 | JASSXV000000000 |
| MCD39 | 535 | 4308967 | 13654 | JASSXU000000000 |
| MCD4 | 564 | 4157130 | 12516 | JASSXT000000000 |
| MCD40 | 566 | 4155543 | 12105 | JASSXS000000000 |
| MCD41 | 1253 | 4076333 | 5109 | JASSXR000000000 |
| MCD42 | 411 | 4279617 | 20032 | JASSXQ000000000 |
| MCD43 | 497 | 4149642 | 15362 | JASSXP000000000 |
| MCD44 | 429 | 4128292 | 16479 | JASSXO000000000 |
| MCD45 | 463 | 4182233 | 16006 | JASSXN000000000 |
| MCD46 | 471 | 4150886 | 16842 | JASSXM000000000 |
| MCD47 | 489 | 4035931 | 13786 | JASSXL000000000 |
| MCD48 | 465 | 4029815 | 15734 | JASSXK000000000 |
| MCD49 | 577 | 4169516 | 12112 | JASSXJ000000000 |
| MCD5 | 374 | 4105788 | 21501 | JASSXI000000000 |
| MCD50 | 407 | 3952144 | 19262 | JASSXH000000000 |
| MCD51 | 480 | 3939707 | 13719 | JASSXG000000000 |
| MCD52 | 456 | 4057190 | 14412 | JASSXF000000000 |
| MCD53 | 560 | 4274754 | 13547 | JASSXE000000000 |
| MCD54 | 356 | 3975653 | 22240 | JASSXD000000000 |
| MCD55 | 498 | 4282038 | 16904 | JASSXC000000000 |
| MCD57 | 407 | 4106181 | 17492 | JASSXB000000000 |
| MCD58 | 405 | 4064543 | 19423 | JASSXA000000000 |
| MCD6 | 343 | 3978591 | 18986 | JASSWZ000000000 |
| MCD60 | 363 | 4079390 | 19063 | JASSWY000000000 |
| MCD61 | 496 | 4014710 | 13264 | JASSWX000000000 |
| MCD64 | 639 | 4121503 | 10963 | JASSWW000000000 |
| MCD66 | 316 | 3982199 | 25317 | JASSWV000000000 |
| MCD67 | 426 | 4055044 | 16663 | JASSWU000000000 |
| MCD68 | 374 | 4159922 | 22748 | JASSWT000000000 |
| MCD7 | 401 | 4095006 | 20135 | JASSWS000000000 |
| MCD70 | 275 | 3994138 | 27814 | JASSWR000000000 |
| MCD71 | 365 | 4210622 | 22305 | JASSWQ000000000 |
| MCD72 | 416 | 4097578 | 20882 | JASSWP000000000 |
| MCD73 | 346 | 4015022 | 20750 | JASSWO000000000 |
| MCD74 | 382 | 4008155 | 21943 | JASSWN000000000 |
| MCD76 | 408 | 4003710 | 20083 | JASSWM000000000 |
| MCD77 | 636 | 3940866 | 10245 | JASSWL000000000 |
| MCD78 | 497 | 4081941 | 15084 | JASSWK000000000 |
| MCD79 | 454 | 4038762 | 15822 | JASSWJ000000000 |
| MCD8 | 296 | 4005250 | 22779 | JASSWI000000000 |
| MCD80 | 429 | 4152765 | 18004 | JASSWH000000000 |
| MCD81 | 310 | 3968343 | 24439 | JASSWG000000000 |
| MCD83 | 516 | 4134709 | 13567 | JASSWF000000000 |
| MCD9 | 443 | 4022708 | 15681 | JASSWE000000000 |

Table 2: Reference sequences for gene content analysis

| **Reference Gene Sets** | **Accession** |
| --- | --- |
| **Transposons** | |
| Tn*916* | KM516885.1 |
| Tn*5397* | AF333235.1 |
| Tn*5398* | AF109075.1 |
| Tn*B1230* | AM749838.1 |
| Tn*4453a* | AF226276.1 |
| Tn*6218* | HG002396.1 |
| Tn*6164* | gi_291482100 |
| Tn*6194* | HG475346.1 |
| Tn*6215* | KC166248.1 |
| Tn*1549* | AF192329.1 |
| Tn*1549* (vanB region) | AF192329.1 |
| Tn*1549*-like (C. difficile) | KU558763.1 |
| Tn*1549*-like (C. difficile - vanB region) | KU558763.1 |
| Tn*6189* | MK895712.1 |
| Tn*6218* | HG002387.1 |
| Tn*6215* | KC166248.1 |
| Tn*5398* | AF109075.2 |
| Tn*6086* | HM636636.1 |
| **Phage** | |
| CDHM1 | HG531805.1 |
| ϕC2 | NC_009231.1 |
| ϕCD119 | NC_007917 |
| ϕCD27 | NC_011398.1 |
| ϕCD38-2 | NC_015568.1 |
| **Plasmids** | |
| pCD6 | AY350745.1 |
| pCD630 | NC_008226.2 |
| pDLL3026 | PRJNA445766 |
| pCDBI1 | FN668942.1 |
| **Toxins** | |
| PaLoc – 630 (*tcdA, tcdB)* | NC_013315.1 |
| CdtLoc – 630 (truncacted *cdtA, cdtB*) | NC_013315.1 |
| CdtLoc – CD196 (*cdtA, cdtB*) | EF581852.1 |
